# How does digital economy drive industrial structure upgrading: An empirical study based on 249 prefecture-level cities in China

**DOI:** 10.1371/journal.pone.0277787

**Published:** 2022-11-17

**Authors:** Tingli Wu, Wei Shao

**Affiliations:** 1 School of Economics, Zhejiang University of Finance & Economics, Hangzhou, Zhejiang, China; 2 School of modern finance, Jiaxing Nanhu University, Jiaxing, Zhejiang, China; Universiti Malaysia Sabah, MALAYSIA

## Abstract

China is in a critical period of economic restructuring and optimization, and the vigorous development of the digital economy plays a vital role in the transformation and upgrading of the industrial structure. Using the panel data of 249 prefecture-level cities from 2011 to 2018, this study empirically investigates the relationship and mechanism between digital economy and industrial structure upgrading. The results show that the digital economy significantly promotes the upgrading of the industrial structure, and this conclusion is still valid after robustness tests such as selecting historical data as instrumental variables. The analysis of the mechanism of action shows that the improvement of labor efficiency and the promotion of technology spillover are the important mechanisms of the digital economy to promote industrial structure upgrading. Finally, the study of regional differences shows that the eastern region has the most obvious promotion effect of digital economy development, the central region is the second, and the western region has the least impact. The research here promotes the understanding of the motivation of industrial structure upgrading and the effect, mechanism, and regional differences of the digital economy enabling the development of a modern industrial system.

## Introduction

The digital economy is a new type of economy based on the innovation and integration of information and communication technologies such as cloud computing, big data, artificial intelligence (AI), Internet of Things, Block Chains, and mobile Internet. This economy can drive substantial changes in social production methods and increase production efficiency. The 14th Five-Year Plan for National Economic and Social Development and the Long-Range Objectives Through the Year 2035 proposed “embracing the digital age, activating the potential of data elements, promoting the construction of a network power, accelerating the construction of a digital economy, digital society, and digital government. Digital transformation drives the transformation of the production mode, lifestyle, and the governance mode.” The digital economy has gradually become the main engine of growth. From 2005 to 2019, the scale of China’s digital economy has risen from 2.6 trillion yuan to 35.9 trillion yuan, and its share of GDP has also risen from 14.2% to 36.2%. In 2020, the Chinese digital economy continued to vigorously develop. The scale of the digital economy was 39.2 trillion yuan, increasing 3.3 trillion yuan from last year. China’s digital economy has gradually become an important component and growth force of the national economy through maintaining high speed growth. Simultaneously, as China’s economy enters a transition period, it is urgent to overcome obstacles to economic structural transformation and upgrading. The fundamental task is the transformation and upgrading of industrial structure, and the digital economy is regarded as an important support to promote industrial transformation and upgrading. Therefore, how to effectively release the digital economy’s driving force for the transformation and upgrading of China’s industrial structure has recently become an action topic widely discussed by the government and all social sectors.

Can the digital economy solve the practical problems of China’s industrial structure? Can it improve the level of industrial informatization? What is the internal mechanism of the digital economy’s effect on industrial structure? This is the focus of this study. To address these questions, although the Internet has made great achievements in real life, and the digital economy has gradually become an important part of the national economy, accurate empirical evaluations of the digital economy’s role in industrial upgrading remain scarce. There are only two kinds of relevant literature: one is about the theoretical elaboration of the realization path; the other is about the influence of informationization and intelligentization on industrial structure. To answer the above questions, it is necessary to conduct empirical research on the basis of sorting out relevant theories and combining with the actual situation in China, which also provides opportunities for marginal contribution in this paper. Based on the panel data of 249 prefecture level cities in China from 2011 to 2018, this research explores the influence mechanism of the digital economy’s promotion of industrial structure adjustment. This paper is organized as follows: the first section is a literature review. The second section provides a mechanism analysis and research hypothesis. The third section describes the methodology, and the fourth shows the empirical results. The fifth section concludes and and provides policy recommendations.

## Literature review

The digital economy represents new economic forces and advanced productivity. It constitutes an inevitable trend of social and technological progress. However, the development of digitalization in China remains in the early stage. Related research mainly focuses on the impact of informatization and intellectualization on the industrial structure. Little research exists on the "influence of digital economy on the optimization and upgrading of industrial structure." Research closely related to this study can be divided into the following three categories:

The first category is research on the connotation and measurement of the digital economy. The concept of digital economy appeared with the rise of the Internet. Tapscott formally posited the concept of digital economy [1]. Digital economy is an economic activity driven by digital technology [2]. The digital economy consists of two parts: one is the digital economy industry itself, and the other is the new output brought by the integration of digital technology into traditional industries. The White Paper on China’s Digital Economy Development believes that the digital economy is based on digital knowledge and information as the key production factors, digital technology as the core driving force, modern information networks as an important carrier that continuously improves the digitalization level, networking, and the intellectualization of the economy and society. Regarding measuring the digital economy, the existing methods can be generally divided into two categories: one is the direct method, which directly estimates the digital economy’s scale with the help of the relevant methodology of national economic accounting. The most representative examples are the OECD’s digital economy research framework and the US Bureau of Economic Analysis’s accounting system. The other is the indirect method, that is, to construct an index system based on multiple dimensions of digital economy connotation. The "Internet Plus" digital economy index jointly constructed by Tencent and other institutions has been used for reference by many scholars such as Wang Binyan and Jiang Song [3, 4]. In addition, Zhao, et al. [5], HanLu [2] and other scholars included digital financial inclusion in the index system as an important component of the development of digital economy.

The second category is research on the digital economy’s impacts. Most of this literature focuses on the digital economy’s impact on macro and micro economies. At the micro level, digital economy reduces the cost of economic activities, including search and replication costs [6, 7] as well as transportation [8, 9], tracking [10], and verification costs [11, 12], promoting enterprise green technology innovation [13], household consumption upgrading [14], and enterprise digital transformation [15]. At the macro level, the digital economy based on Internet technology has a significant impact on China’s total factor productivity [16], inclusive growth [17] and regional integration level [18].

The third category is research on the relationship between digital economy and industrial structure transformation and upgrading. Most of the early studies were conducted from the perspective of digital industrialization or industrial digitization. Li Yiming believes that the digital economy has gradually become an important part of the new economy [19]. The digital economy plays an important role in the formation of new industries and business formats. Advancing the development of the digital economy plays an important role in the transformation and upgrading of industry. Zhang Yuzhe explained the important role of the digital economy in promoting the industrial structure, and introduced the digital economy’s development ideas, main principles, and main tasks in promoting industrial upgrading. The digital economy can not only broaden market scope by reducing transaction costs and improving transaction efficiency, but also improve industrial quality, optimize industrial structure [20], and enhance efficiency of factor resource allocation [21]. In addition, the digital economy has the characteristics of high innovation frequency, high degree of influence, and strong spillover [22], which is conducive to enterprises learning new technologies and business models [23].

The existing research mainly has the following shortcomings. First, the literature offering a comprehensive view of the relationship between the digital economy and the industrial structure is not rich; in particular, empirical research is scarce. Secondly, the existing research did not analyze the mechanism of how the digital economy influences the upgrading of China’s industrial structure. Compared with the existing literature, the main contributions of this study are the following: first, it analyzes the internal mechanism of the digital economy influencing the industrial structure from both direct effects and intermediary effects; second, build a more comprehensive digital economy indicator measurement system and measure the digitial economy’s development level in 249 prefecture-level cities; third, using a variety of measurement methods and orefecture-level city data, test the degree of influence of the digital economy on industrial upgrading. This can provide more convincing empirical evidence for the digitial economy’s impact on the transformation and upgrading of the industrial structure.

## Mechanism analysis and research hypothesis

The new round of scientific and technological revolution and industrial transformation are advancing vigorously, and the industrial structure is evolving rapidly and displaying new characteristics. The most notable one is the vigorous development of the digital economy having a major impact on industrail evolution. According to the Industrial Structure Theory [24], the upgrading of industrial structure is a process: original elements or resources will shift from traditional industries with lower allocation efficiency to knowledge-intensive or technology-intensive industries with higher allocation efficiency, or shift from low-value-added industries to high-value-added industries. This change has led to the continuous increase in the share of modern industries and upgrading of the industrial structure. Digital economy is a new economic paradigm with data as the key element, the information network as the carrier, and digital industrialization and industrial digitization as the value. The digital economy stimulates the development of emerging industries and transforms traditional industries through digital industrialization and industrial digitization. It constantly reshapes the basic form of the industrial structure [25], and promotes its upgrading.

According to experience and the fact that the digital economy’s rapid growth has driven national economic development in recent years, the challenges brought by a measurement method, and the objective needs of building a new system of high-quality, efficient, structural optimization, and competitive modern industries, this study reconstructs the digital economy indicator system by combing and summarizing relevant literature. Using the aspects of labor productivity and technology spillovers, the following theoretical framework is constructed to discuss the impact of the digital economy on industrial structure upgrading.

### The effect of labor efficiency enhancement

The upgrading of industrial structure means that production factors, such as capital, labor and data, can effectively flow to efficient industries. Marketization has realized the free flow and reasonable and efficient factor allocation, thereby adjusting the economic structure, improving economic efficiency and promoting the transformation and upgrading of the industrial structure [26]. The important factors affecting China’s industrial transformation and upgrading include factor marketization and modernization of government regulatory capacity, which have a significant positive effect on the upgrading of industrial structure [27]. The improvement of information infrastructure plays a significant role in promoting the industrial optimization and upgrading and is affected by the completeness of the factor market and legal environment [28]. The digital economy has strengthened the three ways to promote economic growth, namely, optimizing factor allocation, upgrading factor combination, and improving production efficiency. The application of digital information technology can bring more reasonable factor allocation to the market, thus promoting high-quality economic development [29].

At present, digital technology transformation has improved productivity informatization, digitization, and intelligence. It breaks the shackles of the traditional labor market from many aspects such as technological and economic models, and greatly improves labor efficiency. Theoretically, digital economy development can improve labor efficiency from two aspects. On the one hand, such development generally improves the allocation efficiency of China’s labor resources. Time and space barriers in the original labor model have been broken. This can provide more choices for workers’ employment freedom. Digital economy promotes the flow of labor and other factors to more efficient industries and improves labor efficiency in general [30]. On the other hand, applying digital technology means that enterprises can use it to process a large amount of non-standard information. Enterprises can reasonably allocate labor resources and improve output efficiency. Therefore, digital economy can improve labor efficiency by reshaping the allocation of labor resources and improving labor quality.

On the premise of free flow of production factors, additional high-quality labor, capital and other production factor resources are attracted to high value-added industrial sectors, whose importance continue to rise. This transfer promotes the upgrading of traditional industries and the emergence of new enterprises [31]. On the other hand, companies are more inclined to choose high-productivity labor. This choice will force workers to continuously improve their quality, and this can improve an industry’s overall labor quality. This choice will further attract additional high-quality people and capital to this industry, forming a human capital agglomeration effect. Therefore, this article believes that the digital economy can promote industrial upgrading through the labor efficiency mechanism.

### The effect of technology spillover

Technological progress will change the types and proportions of production factors and promote productivity by reconstructing the original division of labor and the cooperation system. The digital economy brings new production technologies and business models. Digital technology, such as AI, is a basic and universal technology that comprehensively affects all industries in an economy and the output level of all sectors through technology spillovers and other channels [32–34]. In theory, digital economy development can produce technology spillover effects from two aspects. On the one hand, it has the characteristics of high knowledge density, such as the Internet, AI, and 3D printing. These fields have concentrated a large amount of R&D resources, with high R&D intensity and strong innovation. Simultaneously, the digital economy proliferates, and traditional industries can learn new digital economy technologies, thereby promoting technological innovation in traditional industries. In general, the digital economy has created innovation incentives for traditional industries through diffusion effects. On the other hand, the digital economy has a demonstrative effect on the traditional economy. The digital industry has strong competitiveness to promote the transformation of traditional industry enterprises and improve service quality. The theoretical research results of D’Aspremont and Jacquemin show that the relationship between enterprises is not perfect competition. They will also cooperate in R&D. This cooperation sharing of R&D costs and results can stimulate the positive effects of technology spillovers. The research report "Digital Spillover-Measuring the Real Impact of the Digital Economy" jointly issued by Huawei and the Oxford Economics pointed out that the digital economy mainly achieves spillover through three channels: internal learning practices, intra-industry competition effects, and cross-industry supply chain effects.

The endogenous economic growth theory emphasizes the role of knowledge and technological research in economic growth. In the early stage, the digital economy has a technological spillover effect on the traditional economy, which helps it carry out technological learning and digital transformation. Simultaneously, there is a competitive relationship between the digital and traditional economies. Under the impact of disruptive innovation in the digital economy, the traditional economy may be squeezed out before it has completed its transformation. The role of digital economy in promoting knowledge and technology spillover is conducive to improving the level of firms’ technological innovation and competitiveness, accelerating the transformation of an industry into a technology-intensive industry and promoting the upgrading of the industrial structure [35]. Therefore, we speculate that the digital economy can effectively promote the upgrading of industrial structure through technology spillover effects.

The above theoretical analysis shows that the development of the digital economy can not only directly promote the continuous optimization and upgrading of industry through the evolution of the factor structure, but also by improving labor efficiency and promoting technology spillovers. To verify the above theoretical analysis, this study proposes the following two hypotheses.

**Hypothesis 1: The digital economy promotes the upgrading of local industrial structure**.

**Hypothesis 2: The digital economy promotes the transformation of industrial structure by improving labor efficiency and promoting technology spillovers**.

## Methodology

### Model

To test the impact of digital economy development on industrial structure upgrading, this study sets the basic model of panel data as follows:

$${TH}_{it}=\alpha_{0}+\alpha_{1}{DEII}_{it}+\alpha_{2}{ZG}_{it}+\lambda_{i}+\delta_{t}+\varepsilon_{it}$$ (1)

In Formula (1), *TH*_*it*_ represents the industrial structure upgrading of the explained variable, *DEII*_*it*_ represents the development level of the digital economy, *ZG*_*it*_ represents the control variable. Subscript i indicates the city, t indicates the year, *δ*_*t*_ and *λ*_*i*_ represent time effect and city effect, respectively; *ε*_*it*_ represents random perturbation term. *α*_1_ represents the impact of the development of digital economy on the leap of industrial structure; *α*_2_ is the coefficient vector of the control variable. Control variable *ZG*_*it*_ mainly includes the level of economic development of each region, the level of technological innovation, transportation infrastructure, human capital, foreign investment, and marketization.

### Variables and data

#### Industrial structure upgrading

The industrial structure upgrading index is the explanatory variable of this study. This research draws lessons from the common practice of academic circles: to reflect the height of industrial structure by the proportional relationship of various industries, and to use the proportion of output value of the second and third industries as the measure of the height of industrial structure according to Clark’s law [24]. Set the indicator to *TH*_*it*_ = *Y*_3_/*Y*_2_, where, *Y*_3_ represents the output value of the tertiary industry and *Y*_2_ represents the output value of the secondary industry. The higher the proportion of the tertiary industry’s output value in the secondary industry’s output value, the higher the jump level of the industrial structure and the greater the value of TH.

#### Development level of the digital economy

Referring to the comprehensive development index of digital economy constructed by Zhao, et al. [5], combined with the availability of data at the prefecture level and the period’s span, this study measures the digitial economy’s development level from two aspects: Internet development and digital financial inclusion. As shown in Table 1, the Internet development level is measured by four indicators: Internet penetration rate, Internet related output, number of mobile phone users, and relevant employees, which respectively correspond to the number of Internet broadband access users among 100 people, total telecom services per capita, number of mobile phone users among 100 people, and the proportion of employees in computer services and software industry in employees in urban units. Digital financial inclusion is measured by the China Digital inclusive financial index, which is jointly prepared by the digital financial research center of Peking University and Ant Financial Services Group [36]. Through the principal component analysis method, the data of the above five indicators are standardized and dimensionally reduced to obtain the digital economic comprehensive development index (represented by the symbol DEII).

**Table 1:** Evaluation index system of digital economy development.

Grade 1	Grade 2	Grade 3	Index attribute
Internet development level	Internet penetration rate	Number of Internet broadband access users per 100 people	+
	Internet related output	Total telecom services per capita	+
	numbers of mobile phone owners	Number of mobile phone users per 100 people	+
	Relevant employees	Proportion of employees in computer service and software industry in employees in Urban Units	+
Inclusive level of digital finance	Digital Inclusive Finance	China Digital Inclusive Financial Index	+

### Control variable

To more comprehensively analyze the role of digital economy development in industrial structure upgrading, we must also set relevant control variables that may have an impact, as follows: regional economic development level (LNAGDP), expressed by logarithm of per capita GDP; technological innovation level (TIS), expressed by the logarithm of the number of patent applications accepted by prefecture level cities. The level of foreign investment (FDIG) is measured by the ratio of the amount of foreign investment actually utilized by local cities to GDP converted according to the RMB exchange rate over the years; the level of transportation infrastructure (IFS) is measured by the per capita road area of prefecture level cities; the level of human capital (HC) is expressed by the proportion of the number of ordinary colleges and universities in prefecture-level cities in the total population of the region; the market-oriented index (MAKI) is measured by referring to the market-oriented index of Dai Kuizao and Liu Youjin [31].

### Data description

This study conducts an empirical analysis on 249 cities in China from 2011 to 2018. The China Digital Inclusive Finance Index used here comes from the digital finance research center of Peking University [36]. The patent application data reflecting the level of technological innovation comes from China’s patent database. Other original data are from China Statistical Yearbook and China Urban Statistical Yearbook. The relevant information of main variables is shown in Table 2. The mean value of Digital Economic Development (DEII) is -0.020, the maximum value is 7.962, the minimum value is -1.311, and the standard deviation is 1.489, showing the characteristics of "small mean and large standard deviation," indicating that the development level of digital economy in different regions differs substantially. To eliminate the possible adverse effects of outliers, 1% tail reduction is applied to all continuous variables.

**Table 2:** Summary statistics.

Variable	N	Mean	Std. Dev.	Min.	Max.
Industrial structure upgrading (TH)	1,992	0.923	0.458	0.313	3.200
Development level of digital economy (DEII)	1,992	-0.020	1.489	-1.311	7.962
Regional economic development level (LNAGDP)	1,992	1.467	0.542	0.335	2.737
Technological innovation level (TIS)	1,992	6.562	1.812	2.833	10.803
Transportation infrastructure level (IFS)	1,992	12.835	6.829	1.900	39.783
Human capital level (HC)	1,992	0.088	0.011	0.075	0.123
Foreign investment level (FDIG)	1,992	1.742	1.625	0.022	7.467
Marketization index (MAKI)	1,992	6.994	1.495	4.370	10.810
Labor efficiency (LAB)	1,992	35.844	18.550	7.958	106.361
Technology spillover (LNTS)	1,992	7.930	0.697	6.801	10.532

## Results

### Benchmark estimation results

Taking industrial structure upgrading (TH) as the explanatory variable and digital economy development level (DEII) as the core explanatory variable, this study conducts an empirical study on the relationship between digital economy and industrial structure jump. According to the Hausman test results, this study uses the two-way fixed effect model to estimate Eq (1), and the effects of serial correlation and heteroscedasticity on the estimation results are alleviated with standard errors clustered at the city level.

Columns (1) and (2) in Table 3 report the Pooled OLS regression results of local industrial structure upgrading. The results show that at the 1% significant level, the influence coefficient of digital economy development level (DEII) on industrial structure upgrading (TH) is 0.118, after controlling the important variables such as the local technological innovation, foreign investment, transportation infrastructure, human capital and marketization levels, the regression coefficient of digital economy development on the jump of industrial structure remains significantly positive, and the coefficient value is 0.109. Columns (3) and (4) report the regression results of fixed effects. The coefficient value of digital economic development level (DEII) is always significantly positive at the 1% level. Column (4) shows the result of the fixed effects model. The digital economy has a significant positive effect on industrial structure, with an impact coefficient of 0.084, and is significant at the 1% level, which indicates that in China’s digital economy, every one percentage point increase in the level of development directly increases the industrial structure by 0.084 percentage points, which proves that the digital economy plays an important role in increasing industrial structure upgrading, that is, the development of digital economy significantly promotes the upgrading of China’s industrial structure, verifying **Hypothesis 1**.

**Table 3:** Impact of digital economy on industrial structure upgrading.

	(1)	(2)	(3)	(4)
Variable	TH	TH	TH	TH
DEII	0.118*** (0.0064)	0.115*** (0.0085)	0.258*** (0.0098)	0.099*** (0.0109)
Constant	0.925*** (0.0095)	-0.687*** (0.1115)	0.927*** (0.0040)	-1.607*** (0.1318)
Control variables	NO	YES	NO	YES
Estimation method	Pooled OLS	Pooled OLS	Fixed effect	Fixed effect
Fixed year	NO	NO	YES	YES
Urban fixed	NO	NO	YES	YES
Observed value	1,992	1,992	1,992	1,992
R-squared	0.147	0.270	0.286	0.517
Number of reg	249	249	249	249

Note: (1) * * *^,^ * *^,^ * respectively indicate that the statistical value is significant at the significance level of 1%, 5% and 10%, respectively. (2) The values in parentheses are standard errors. Ibid., Tables 4–7.

### Endogenous problems

The model (1) in this study may have endogenous problems, that is, the upgrading of industrial structure and digital economy development may be affected by a series of unobservable factors such as knowledge, technological innovation, and demand structure, resulting in biased estimation of regression coefficients. This study attempts to find appropriate instrumental variables to further alleviate the endogenous problem, so as to identify the net effect of the development of digital economy on the upgrading of local industrial structure. This research draws on the methods of Zhao, et al. [5] and Huang Qunhui, et al. [37], and uses the historical data of Posts and Telecommunications in 1984 as the instrumental variable of the digital economic development index to alleviate the endogenous problem. On the one hand, modern communication technologies such as Internet and digital technology are developed from traditional communication technologies such as fixed telephone. Factors such as the level of local historical telecommunications infrastructure and usage habits will affect the subsequent development of the digital economy. Therefore, the historical situation of post and telecommunications has a high correlation with the development of digital economy; On the other hand, with the decline in use of traditional communication facilities such as fixed telephone, its influence on the upgrading of industrial structure gradually disappears, which satisfies the exogeneity. It should be noted that the samples studied here are panel data, and the number of fixed phones per 100 people in each city in 1984 is cross-sectional data, which cannot be directly used for the econometric analysis of panel data. Therefore, referring to the processing method of Nunn and Qian [38], a time series variable is introduced to construct the panel tool variable. In this paper, the number of Internet users in China in the previous year and the number of telephones per 10000 urban people in 1984 are used to construct the interactive term (LNTE) as the instrumental variable of the urban digital economy development index.

Table 4 reports the estimation results of the fixed effect two-stage least squares method (2SLS) after the introduction of instrumental variables. Columns (1) and (3) show that before and after controlling for the explanatory variables, the coefficients of LNTE are 1.768 and 1.834, respectively, and the results are both significantly positive at the 1% level, indicating that the correlation hypothesis of instrumental variables is met. The results in column (2) and (4) show that after considering endogeneity, the coefficients of DEII are 0.509 and 0.469, respectively, and the results are both significantly positive at the 1% level, indicating that the effect of digital economy development on upgrading industrial structure remains valid. The coefficient estimation results of each explanatory variable have no significant difference in impact direction and significance, which verifies that the previous conclusions are robust and the important impact of the development of digital economy on the jump in local industrial structure.

**Table 4:** Estimation results of instrumental variables.

	(1)	(2)	(3)	(4)
	Step1	Step2	Step1	Step2
Variable	DEII	TH	DEII	TH
LNTE	1.768*** (0.0418)	-	1.701*** (0.1037)	-
DEII	-	0.509*** (0.0161)	-	0.593*** (0.0439)
Control variables	NO	NO	YES	YES
Estimation method	2sls	2sls	2sls	2sls
Observed value	1992	1992	1992	1992
Kleibergen-Paap rk LM	-	883.404 [0.0000]	-	233.624 [0.0000]
Kleibergen-Paap rk Wald F	-	1792.360 {16.3800}	-	268.817 {16.3800}
R2	0.336	0.017	0.434	0.218

In addition, for the test of "insufficient identification of instrumental variables" of the original hypothesis, the LM statistics P values of Kleibergen-Paap rk are all 0.000, which significantly rejects the original hypothesis. In the test of weak identification of instrumental variables, the Wald F statistic of Kleibergen-Paap rk is 338.640, which is greater than the critical value (16.38) at the 10% level of the Stock-Yogo weak identification test, indicating that there is no problem of weak instrumental variables, and the regression results of the second stage show that the coefficient of endogenous variable DEII does not change significantly under the condition of controlling relevant variables. Therefore, it indirectly shows that the estimated variables meet the exclusive constraints, that is, meet the exogenous requirements. In general, after considering the endogenous problem, the conclusions of this study remain valid.

### Robustness test

#### Re-select the measurement index of industrial structure upgrading

Since it is also a commonly used measurement index in academic circles to reflect the upgrading of industrial structure by using the proportional relationship of various industries [24], the following formula is used for reference to calculate the upgrading of industrial structure (expressed by the symbol SH), that is, SH = Y_3*t*_/*Y*_*t*_, where Y_3t_ represents the industrial added value of the tertiary industry at t, and Y_t_ is the total industrial added value. If SH is rising, it indicates that the industrial structure is advancing in the direction of high technology, knowledge, and added value, and the industrial structure is upgrading. If the influence coefficient of digital economy development on SH is significantly positive, it shows that its development promotes the upgrading of industrial structure.

The regression results in column (1) of Table 5 show that the estimated coefficient of digital economy development remains significantly positive (0.007), indicating that the development of the digital economy promotes the transformation and upgrading of industrial structure, which is consistent with the benchmark regression results. This shows that after considering different measurement methods of industrial structure upgrading, the main conclusion of this study is robust.

**Table 5:** Robustness test results.

	(1)	(2)
Variable	SH	TH
DEII	0.013*** (0.0035)	0.056*** (0.0099)
FD	-	25.326*** (1.1742)
Constant	0.290*** (0.0426)	-1.296*** (0.1179)
Control variables	YES	YES
Estimation method	Fixed effect	Fixed effect
Fixed year	YES	YES
Urban fixed	YES	YES
R-squared	0.094	0.620
Observed value	1,992	1,992

#### Missing variable method

A large number of studies have shown that financial development is a necessary means and main driver in promoting industrial structure optimization and upgrading [39]. Therefore, this study further tests whether financial development has an impact on the empirical results [40]. Referring to the practices of Shen, et al. [41] and Lv Chaofeng [42], this study uses the ratio (FD) of deposit and loan balance at the end of the year to GDP to measure the level of financial development. The results in column (2) of Table 5 show that after considering the impact of financial development level, both the estimated coefficient and the significance level are consistent with the benchmark regression results.

#### Impact mechanism analysis

On the basis of Hypothesis 1, this section uses the intermediary effect model to test the labor efficiency and technology spillover effects so as to more deeply understand the internal law of digital economy affecting the upgrading of industrial structure. This study introduces the mediating effect model for research, and the specific mediation effect model is set as follows:

$${TH}_{it}=\alpha_{0}+\alpha_{1}{DEII}_{it}+\alpha_{2}{ZG}_{it}+\lambda_{i}+\delta_{t}+\varepsilon_{it}$$ (2)

$${MG}_{it}=\beta_{0}+\beta_{1}{DEII}_{it}+\beta_{2}{ZG}_{it}+\lambda_{i}+\delta_{t}+\varepsilon_{it}$$ (3)

$${TH}_{it}=\gamma_{0}+\gamma_{1}{DEII}_{it}+\gamma_{2}{MG}_{it}+\gamma_{3}{ZG}_{it}+\lambda_{i}+\delta_{t}+\varepsilon_{it}$$ (4)

*MG*_*it*_ is an intermediary variable, including two proxy variables (labor efficiency and technology spillover). In Eq (3), *β*_1_ reflects the impact of digital economy on intermediary variables, *β*_2_ is the coefficient vector of the control variable, and the coefficient in Eq (4), *γ*_2_ indicates the influence effect of intermediary variables on industrial structure upgrading, and other symbols have the same meaning as Formula (1) above. If the labor efficiency or technology spillover effects are the intermediary effect of digital economy on industrial structure upgrading, then *β*_1_ and *γ*_2_ should be positive or negative.

Regarding the index selection of intermediary variables, labor efficiency refers to the output level of unit labor input. This study uses the ratio of industrial added value to total employment in each city in that year (expressed by the symbol LAB). The greater the ratio, the higher the labor efficiency. Regarding the technology spillover effect, the rise in technology transaction volume promotes the application and promotion of digital technology, which can better reflect the diffusion and spillover effect of new technology. It is an ideal index to measure technology spillover. This study selects the technology transaction volume (LNTS) of each region as the proxy variable of Technology’s spillover effect.

In this section, we verify Hypothesis 2 according to the recursive model and explore whether the two intermediary effects of labor efficiency effect and technology spillover effect are effective. Table 6 reports the regression results of the fixed effect of Eqs (2)–(4). From the results, we can see that the coefficient of the digital economic development index (DEII) in the first step of the recursive model is significantly positive, the coefficient of labor efficiency effect and technology spillover effect (*β*_1_、*γ*_2_) are significantly positive, and the coefficient of the third step test digital economy development index is also significant, indicating that there are two intermediary effects of digital economy on the upgrading of local industrial structure. Specifically.

**Table 6:** Test tesults of mediating effect.

	(1)	(2)	(3)	(4)	(5)
Step	Step1	Step2	Step3	Step2	Step3
Variable	TH	LAB	TH	LNTS	TH
Step	Step1	Step2	Step3	Step2	Step3
DEII	0.099*** (0.0109)	1.565** (0.7032)	0.096*** (0.0109)	0.156*** (0.0093)	0.002** (0.0009)
Constant	-1.607*** (0.1318)	-4.009 (8.5045)	-1.601*** (0.1311)	3.693*** (0.1130)	-3.895*** (0.1420)
LAB	-	-	0.002*** (0.0004)	-	-
LNTS	-	-	-	-	0.619*** (0.0237)
Control variables	YES	YES	YES	YES	YES
Fixed year	YES	YES	YES	YES	YES
Urban fixed	YES	YES	YES	YES	YES
Observed value	1992	1992	1992	1992	1992
R-squared	0.517	0.078	0.523	0.872	0.654

#### Labor efficiency improvement effect

The estimated results of labor efficiency effects are reported in columns (2) and (3) of Table 6. Column (1) shows the results of the baseline regression; therefore, it is the same as column (4) of Table 3 above and will not be repeated again. Column (2) shows that the influence coefficient of the development level of digital economy on labor efficiency is significantly positive at the level of 1%, with a value of 2.785, indicating that the development of digital economy has significantly promoted labor efficiency improvement. The coefficient value of labor efficiency in column (3) is significantly positive at the 1% level (value is 0.001), indicating that labor efficiency is an important factor promoting the industrial structure upgrading. In addition, the coefficient of digital economy is also significantly positive at the 1% level (value is 0.081). This proves that digital economy can promote the upgrading of the local industrial structure through the transmission channel and mechanism of affecting labor efficiency.

#### Technology spillover effect

It can be seen from the estimation results in column (4) of Table 6 that the impact coefficient of digital economy on the level of technology spillover is significantly positive at the 1% level, with a value of 0.171. This shows that digital economy development not only promotes local technological innovation but also promotes technology spillover. The coefficient of technology spillover in column (5) of Table 6 is significantly positive at the 1% level (value is 0.564), indicating that the improvement of technology spillover promotes the upgrading of local industrial structure, and the coefficient of digital economy is significantly positive at the level of 1% (coefficient value is 0.013). This proves that digital economy can promote the upgrading of local industrial structure by promoting the transmission mechanism of technology spillover.

In summary, the empirical test results of intermediary effect show that digital economy promotes the upgrading of China’s industrial structure by improving labor efficiency and promoting technology spillover, verifying Hypothesis 2.

#### Heterogeneity analysis

Owing to the imbalance of regional economic development in China, there are great differences in economic development level, resource endowment, and industrial structure among cities, which may lead to different effects of the digital economy on industrial structure upgrading in different cities.

To verify whether there are regional differences in the digital economy’s impact on the upgrading of industrial structure, we divide the country into eastern, central, and western regions. Set the dummy variables ER, MR, and WR. ER means that if the region belongs to the eastern region, the value is 1; otherwise, the value is 0. If it is the central region, MR is 1, otherwise it is 0. If it is the western region, WR is 1, otherwise it is 0. Columns (1), (2) and (3) of Table 7 report the estimation results of fixed effects in the East, West, and central respectively. It can be seen that the significance and influence direction of explanatory variable coefficient are basically consistent with the benchmark model, indicating that the relevant results are relatively robust.

**Table 7:** Regional heterogeneity analysis.

VARIABLES	(1)	(2)	(3)
	Eastern region	Western region	Central region
	TH	TH	TH
DEII	0.121*** (0.0243)	0.080*** (0.0133)	0.120*** (0.0243)
Constant	-2.107*** (0.1899)	-0.263 (0.2339)	-1.754*** (0.3613)
Control variables	YES	YES	YES
Observations	776	472	744
R-squared	0.609	0.549	0.460
Number of reg	97	59	93

The results in columns (1), (2) and (3) of Table 7 show that the coefficient values of digital economy in the eastern, western and central regions are significantly positive at the 1% level, with values of 0.116, 0.066 and 0.081 respectively, which shows that the variable symbols and significance of the national overall and subregional regression results remain basically unchanged, indicating that they are relatively stable; that is, digital economy development has promoted the upgrading of industrial structure in various regions of China. In addition, comparing the coefficient values of the three regions, it is found that the promotion of digital economy development in the eastern region is the most obvious, followed by the central region, and the western region has the least impact. It shows that the development of digital economy plays a greater role in promoting the industrial structure of the region with more rapid economic development. The possible explanation for this is: on the one hand, compared with the central and western regions, the eastern region has obvious advantages in cultivating and attracting high-quality talents. Whether in terms of human capital accumulation effect or talent introduction policy, the labor efficiency of the eastern region is higher than that of other regions, and the spillover effect of advanced technology is better, Thus, it can better promote the upgrading of industrial structure. On the other hand, the eastern region’s positive industrial foundation, resource endowment advantages, and the relative maturity of big data, Internet and Internet of things make the coastal areas play a leading role in the upgrading of China’s industrial structure. Driven by the national industrial transfer policy, the eastern region has vigorously developed high-tech industries and high-end equipment manufacturing. As a result, under the similar development level of digital economy, the upgrading degree of industrial structure in the eastern region is higher.

## Conclusion

In the context of China’s economic transformation, the digital economy has become one of the important ways to promote the upgrading of industrial structure. Academic circles have not comprehensively discussed the effect and mechanism in the impact of the digital economy on such transformation and upgrading. For this reason, this study uses panel data of 249 prefecture-level cities from 2011 to 2018 to conduct empirical tests, demonstrates its transmission mechanism from the perspective of labor productivity and technology spillover, and draws the following main conclusions and enlightenments.

First, the empirical results show that the digital economy can significantly promote the transformation and upgrading of industrial structure in China’s prefecture-level cities. Therefore, government departments should attach great importance to the role of digital economy in promoting the upgrading of industrial structure, adhere to long-term development, overall planning, and formulate mid—and long-term digital economy development plans. Simultaneously, it is necessary to clarify the entry point and combination in the development of traditional industries and the digital economy, promote the promotion and application of key core digital technologies, and continue to enhance industrial digitalization and digital industrialization. Digital transformation will drive the transformation of modes of production, lifestyles, and governance as a whole, and promote the upgrading of China’s industrial structure and the real economy’s high-quality development.

Second, the digital economy promotes upgrading of industrial structure primarily through improving labor productivity and technology spillover. Therefore, digital skills should be taken as the starting point to promote supply-side labor market and education reform and increase efforts to cultivate interdisciplinary talents with digital quality. A system of lifelong learning and job training is required to meet the needs of the digital economy development and improve society’s overall understanding and application of it. By contrast, from the perspective of technology spillover, preferential tax policies should be established for enterprises using advanced digital technology to support the application and promotion of advanced digital technology, the application of advanced technology, and the transformation of scientific and technological achievements.

Third, there are obvious regional differences in the digital economy’s impact on industrial structure upgrading. The promotion effect of digital economy development is particularly significant in the eastern region, followed by the central region, and then the western region. Therefore, government departments should reasonably lay out the digital industry space nationwide and promote the coordinated development of the digital economy. There are regional differences in the development of the digital economy, which will become a major factor affecting coordinated regional development. Currently, it is necessary to distribute the digital economy in a balanced way across the country, focus on the digital infrastructure in the central and western regions, bridge the regional gap in the development of the digital industry, and promote the formation of a sound overall development of balanced development and complementary advantages.

## Supporting information

S1 Data (XLSX)
